# Advanced imaging improves the diagnosis of infective endocarditis

**DOI:** 10.12688/f1000research.13791.1

**Published:** 2018-05-29

**Authors:** Daniel Harding, Bernard Prendergast

**Affiliations:** 1 Department of Cardiology, St Thomas’ Hospital, London, UK

**Keywords:** Infective endocarditis, PET, SPECT, Nuclear Imgaing, Cardiology

## Abstract

Infective endocarditis is a heterogeneous condition whose incidence is rising. Despite advances in surgery and diagnostic methods, one-year mortality has not changed and it remains at 30%. Patients with prosthetic valve and intra-cardiac device–related endocarditis are being seen more frequently and this condition is difficult to diagnose with conventional microbiological and imaging techniques. The modified Duke criteria lack sensitivity in this group and should be supplemented with newer imaging techniques, including 18F-fluorodeoxyglucose positron emission tomography (18F-FDG-PET) and single-photon emission computed tomography (SPECT). In this article, we discuss these techniques and their role in the diagnosis of infective endocarditis.

## Introduction

Although infective endocarditis (IE) remains a rare condition worldwide, incidence is rising ^1^ as a combined consequence of the increased number of patients at high risk for the condition ( Table 1) and increased nosocomial infection, possibly exacerbated by the impact of reduced use of antibiotic prophylaxis ^2^. The high-risk population is likely to expand further with increasing rates of invasive procedures, including percutaneous valve replacement and intra-cardiac electronic device (ICED) implantation ^3, 4^. Of equal importance, *staphylococcus* now supersedes *streptococcus* as the most frequent causative pathogen and the average age of affected subjects is rising ^5^. Therefore, patients are more frail and co-morbid and frequently present with more aggressive forms of the disease. These trends seem likely to offset the benefits associated with earlier diagnosis and more aggressive surgical management. As a result, IE has a one-year mortality exceeding 30%, which has remained unchanged over the past 20 years and is higher than that of many common cancers ^6, 7^.

**Table 1.** Risk factors for infective endocarditis.

Prosthetic valve replacement (including percutaneous) Haemodialysis Long-term intravenous catheters Immunosuppression Intravenous drug use Congenital heart disease Implantable cardiac electronic device

Diagnosis of IE remains challenging, requiring a high index of clinical suspicion and appropriate investigation of high-risk patients. Both the European Society of Cardiology (ESC) and the American Heart Association recommend use of the modified Duke criteria ( Table 2) for the diagnosis of IE ^8, 9^. Although these criteria remain useful as a diagnostic aid, they are less sensitive in patients with prosthetic valve endocarditis (PVE), ICED infection ^10^ and IE affecting the right side of the heart. Both major criteria are less sensitive in PVE and ICED: negative blood cultures are common (>20%) and transthoracic echocardiography (TTE) can miss pacemaker lead involvement and PVE in up to 60% of cases ^12, 13^. It is also important to note that the Duke criteria were originally designed as a research tool and should not supplant clinical assessment.

**Table 2.** Modified Duke criteria for the diagnosis of infective endocarditis.

Pathological criteria Microorganisms on histology or culture of a vegetation or intra-cardiac abscess Evidence of lesions: vegetation or intra-cardiac access showing active endocarditis on histology Major clinical criteria 1. Blood cultures positive for infective endocarditis Typical microorganisms consistent with infective endocarditis from two separate blood cultures: - *Staphylococcus aureus*, *viridans streptococci*, *Streptococcus bovis*, HACEK group, or community-acquired *enterococci* in the absence of a primary focus OR Microorganisms consistent with infective endocarditis from persistently positive blood cultures: - At least two positive blood cultures from samples drawn more than 12 hours apart or all of three or most of at least four separate cultures of blood (with first and last sample more than one hour apart) OR Single positive blood culture for *Coxiella burnetii* or phase 1 IgG antibody titre greater than 1:800 2. Evidence of endocardial involvement Echocardiography positive for infective endocarditis - Defined by presence of a vegetation, abscess or new partial dehiscence of a prosthetic valve New valvular regurgitation - Note: increase or change in pre-existing murmur is not sufficient. Minor clinical criteria 1. Predisposition: predisposing heart condition and intravenous drug use 2. Fever: temperature >38°C 3. Vascular phenomena: major arterial emboli, septic pulmonary infarcts, mycotic aneurysm, intra-cranial haemorrhages, conjunctival haemorrhages, and Janeway lesions 4. Immunological phenomena: glomerulonephritis, Osler’s nodes, Roth spots, and rheumatoid factor 5. Microbiological evidence: positive blood culture that does not meet a major criterion or serological evidence of active infection with organism consistent with infective endocarditis Diagnosis of endocarditis is definite in the presence of one pathological criterion, two major criteria, one major and three minor criteria, or five minor criteria. Diagnosis of infective endocarditis is possible in the presence of one major and one minor criterion or three minor criteria. Modified Duke criteria were originally published by Li and colleagues ^11^.

**Figure 1.**
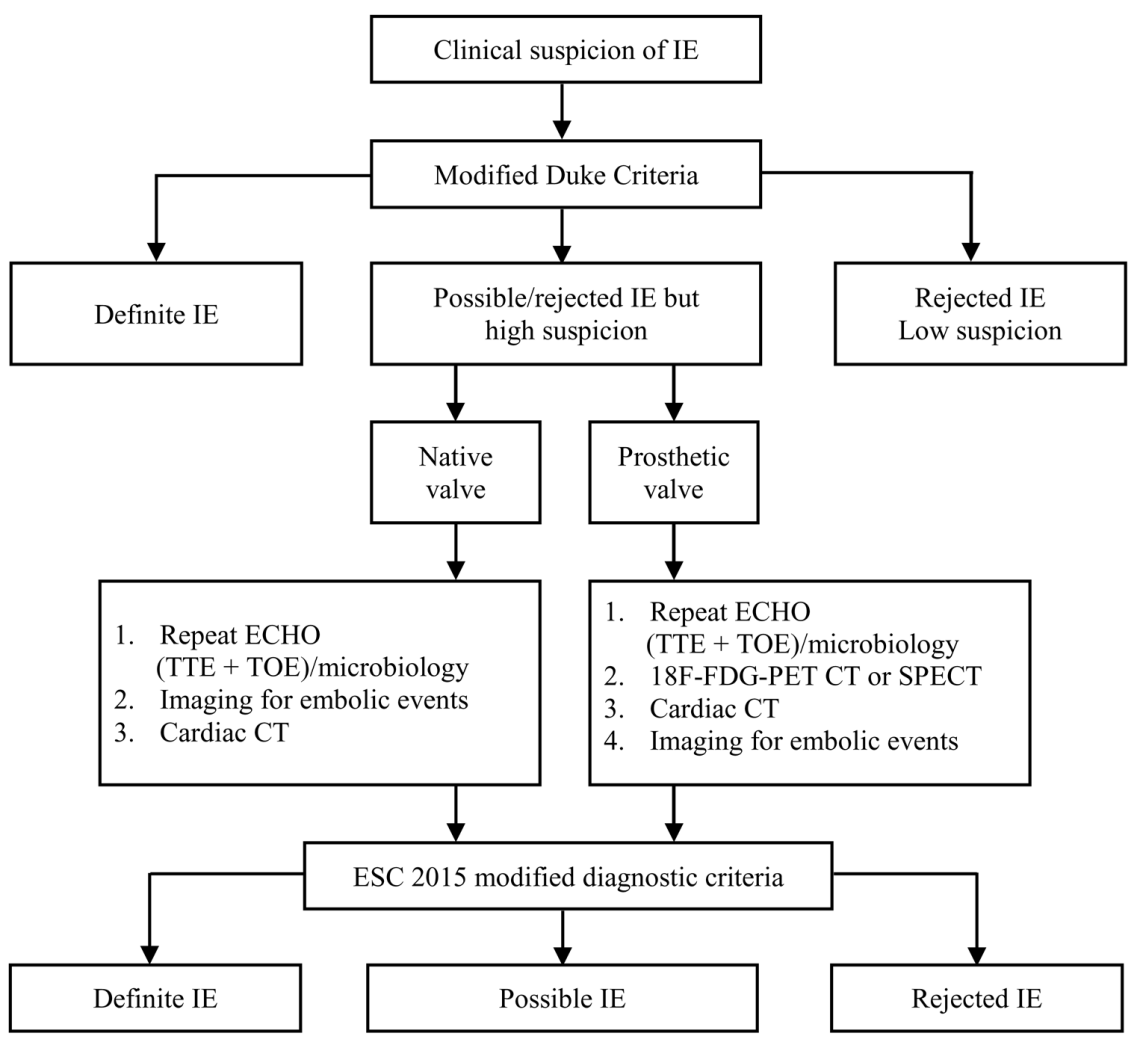
Diagnostic criteria for infective endocarditis. Taken from the 2015 European Society of Cardiology (ESC) guidelines for the management of infective endocarditis (IE) ^9^. 18F-FDG PET, 18F-fluorodeoxyglucose positron emission tomography; CT, computed tomography; ECHO, echocardiogram; SPECT, single-photon emission computed tomography; TOE, transoesophageal echocardiography; TTE, transthoracic echocardiography.

Regardless of the chosen diagnostic guideline, it is likely that a greater proportion of patients with PVE and ICED infection will fall under the ‘possible’ category ( Figure 1) (that is, high-risk patients with some clinical features that do not meet the required cutoff indicated by the modified Duke criteria). Although such patients present a considerable diagnostic challenge, they also stand to benefit the most from the cardiovascular imaging advances that have taken place over the past five years.

## Transoesophageal echocardiography

Transoesophageal echocardiography (TOE) is the gold-standard second-line imaging modality for IE and features prominently in both European and American guidelines. It has a sensitivity of more than 90% for native valve and more than 85% for prosthetic and device-related endocarditis ^13^, is a more accurate tool than TTE for assessing intra- and extra-cardiac lead portions ^14^, and is superior to TTE for detection and characterisation of perforations, abscesses and fistulae ^13^. However, TOE is not without weaknesses. Distinction of active infection from post-operative changes limits its use in patients with suspected PVE who have recently undergone surgery. Furthermore, it can be challenging to differentiate vegetation from thrombi and fibrous strands: about 10% of patients undergoing a TOE for unrelated reasons demonstrate a mass or vegetation with no subsequent diagnosis of IE ^15^. TOE also has limitations in patients with possible ICED endocarditis. Artefact and acoustic shadowing associated with ICED makes it difficult to fully assess the right side of the heart. Indeed, pacemaker leads can be actively infected with no evidence of vegetation on TOE ^14^.

## Three-dimensional transoesophageal echocardiography

Three-dimensional transoesophageal echocardiography (3D-TOE) is carried out with a multi-plane probe incorporating a 3D matrix array. It generates full-volume datasets that can be manipulated to visualise vegetations and defects in planes and at angles that are not possible with conventional TOE ^16^. The technique has particular value in the detection of paravalvular abscess, valve dehiscence, regurgitation and perforation ^9^ and is more specific (but not more sensitive) for the exclusion of IE (up to 100%) when compared with TOE ^17^. 3D-TOE may help to identify patients at increased risk of embolism by providing a more accurate assessment of vegetation size ^18^. Finally, by providing an ‘en-face’ valve view, 3D-TOE may facilitate more accurate surgical planning. Currently, studies supporting the use of 3D-TOE in the diagnosis and management of IE are limited by their small number and size and there have been no studies specifically focused on the use of 3D-TOE in PVE or ICED endocarditis. It is also important to note that the low frame rate of 3D-TOE may impair the detection of small vegetations ^19^. Therefore, the technique should continue to be used as a supplement to conventional TTE/2D-TOE in most patients.

## Multislice computed tomography

Multislice computed tomography (MSCT) is complementary to conventional echocardiography in the diagnosis of IE and indications for its use have been incorporated into the 2014 American College of Cardiology/American Heart Association valvular heart disease guidelines (class IIa, level of evidence B) and the 2015 ESC guidelines on the management of IE ^9, 21^. MSCT uses ECG gating to eliminate movement artefact during the cardiac cycle and reduce radiation exposure to 2–3 mSv per scan ^20^. It is equivalent (and may be superior) to TOE for the delineation of paravalvular anatomy and detection of fistulae and abscess ^22^ and is superior to TOE for the detection of mycotic aneurysms. Use of MSCT may also reduce the need for invasive imaging by characterising the coronary arteries, aortic valve, root and ascending aorta ^23^. It is also less susceptible to prosthetic valve artefact than TOE and therefore is an attractive adjunct in the assessment of suspected PVE. In a group of 28 patients with suspected PVE, the addition of MSCT to routine work-up (including TTE/TOE) resulted in a major diagnostic change in six patients (21%) ^23^. Unlike echocardiographic techniques, MSCT has the potential to consolidate the diagnosis of IE by identifying distal embolic complications if the scan window is extended beyond the thorax. Drawbacks include the need for exposure to ionising radiation, use of iodinated contrast and the lack of large studies comparing the technique with conventional echocardiography.

## Positron emission tomography

18F-fluorodeoxyglucose positron emission tomography (18F-FDG PET) is a relatively new technique more frequently used in the diagnosis and staging of cancer that shows promise for improved assessment of ‘possible’ IE according to the modified Duke criteria. The technique highlights areas of increased glucose metabolism corresponding to active inflammation, which can be mapped to conventional computed tomography (CT) datasets to provide anatomical landmarks. Although early studies generated concerns with regard to low sensitivity in native valve IE (<40%), the technique has demonstrated significant value in the diagnosis of PVE and ICED endocarditis ^24^ (sensitivity of 87% and specificity of 92%) ^25^. Supplementing the diagnostic sensitivity of the modified Duke criteria in suspected PVE and ICED endocarditis increased from 52 to 91% in association with the use of 18F-FDG-PET ^25^, leading to its incorporation into the diagnostic algorithm for high-risk patients with equivocal initial investigations in the 2015 ESC guidelines ^9^. Like MSCT, 18F-FDG-PET can detect distant emboli and evidence of metastatic infection, increasing sensitivity relative to TTE and TOE and identify higher-risk patients—those with more metastatic infection—who may benefit from early surgery ^26^. 18F-FDG-PET has the advantage over MSCT in that it is normal practice to image the whole skeleton rather than just the thoracic cavity. However, as with all imaging techniques, 18F-FDG-PET results must be interpreted with caution. At present, it is not possible to distinguish a sterile, post-operative inflammatory response from infection. Nor can 18F-FDG-PET differentiate thrombi, soft atherosclerotic plaque or foreign body reactions. Given the high mortality associated with device and valve explantation, 18F-FDG-PET data should always be interpreted with caution, especially in the early post-operative phase ^21^. Moreover, sensitivity is low in native valve IE and routine use is not recommended in this group ^27^.

## Single-photon emission computed tomography

Single-photon emission computed tomography (SPECT) is a nuclear technique that measures gamma rays emitted from injected radionucleotides. Multiple 2D plane images are acquired and reconstructed to provide anatomical localisation in 3D. Detection of infection relies on the accumulation of neutrophils in the region of IE and the technique is most sensitive in the acute phase ^21^. There have been relatively few large studies evaluating the utility of SPECT in the diagnosis of IE, but multiple case reports and smaller trials suggest benefit in PVE ^28– 30^. As a result, its use has been incorporated into the 2015 ESC guidelines as an adjunct to repeat TOE/TTE in possible PVE ^9^. SPECT was more specific (100% versus 71%) but less sensitive (64% versus 93%) than 18F-FDG-PET CT in a cohort of 39 patients with suspected PVE ^30^. However, increased specificity in patients who have undergone recent surgery offers advantages: in a group of 131 patients evaluated with SPECT for suspected IE (including those operated within two months), sensitivity was 90% and specificity was 100% ^28^. Conversely, SPECT is more time-consuming than 18F-FDG-PET CT, requires blood handling for preparation of radiopharmaceuticals, and has lower spatial resolution than 18F-FDG-PET CT ^16, 29^. Sadly, current data are mostly limited to native and prosthetic valve endocarditis, so additional work will need to be carried out to establish the full role of SPECT in suspected ICED endocarditis.

## Discussion

The landscape of IE is changing: older and increasingly co-morbid patients with prosthetic material are presenting to hospital with staphylococcal IE that can be difficult to detect by using conventional microbiological and imaging techniques. The modified Duke criteria ( Table 1) are neither sensitive nor specific in this cohort, and more patients are left with a diagnosis of ‘possible’ IE ( Figure 1). Previous guidelines have emphasised the use of 2D-TOE and repeat TTE in this group, but these techniques can also be inadequate to confirm/exclude the diagnosis, especially in the presence of a cardiac device or prosthetic valve. The development of 3D-TOE, MSCT and advanced nuclear imaging (18F-FDG-PET CT and SPECT) facilitates an evaluation pathway in this setting. Patients who are at high risk of IE but have an equivocal or negative TTE (or microbiology) can now progress to diagnostic techniques with high sensitivity and specificity to guide their clinical management. These techniques not only provide local anatomical information in terms of valvular and paravalvular complications but also may strengthen the diagnosis through detection of metastatic infection.

MSCT and 3D-TOE are broadly complementary to conventional diagnostic work-up and may facilitate better surgical planning. In contrast, 18F-FDG-PET CT and SPECT have the potential to determine a diagnosis of IE, even in patients with prosthetic valves and intra-cardiac devices. SPECT is clearly more suitable for patients who have recently undergone surgery as it remains specific, whereas the higher sensitivity and simpler technical aspects of 18F-FDG-PET CT make it an attractive second-line test in patients with a high clinical likelihood of IE and normal or equivocal initial imaging findings. The increasing role and importance of nuclear medicine in the diagnosis and management of IE provide further support for a hub-and-spoke model of service provision, and complex cases are best managed in tertiary reference centres that have the experience and technical resources to offer these techniques routinely.
